# Outflow Graft Obstruction due to Local Aortic Dissection After Implantation of Left Ventricle Assist Device (HeartMate 3)

**DOI:** 10.1097/MAT.0000000000002222

**Published:** 2024-05-09

**Authors:** Lukas Capek, Adrian Thomas Huber, David Reineke, Stephan Dobner, Lukas Christoph Hunziker, Bruno Schnegg

**Affiliations:** From the * Department of Cardiology, Inselspital, Bern University Hospital, University of Bern, Bern, Switzerland; † Department of Radiology, Inselspital, Bern University Hospital, University of Bern, Bern, Switzerland; ‡ Department of Cardiovascular Surgery, Inselspital, Bern University Hospital, University of Bern, Bern, Switzerland.

**Keywords:** acute decompensated heart failure, aortic dissection, HeartMate 3, LVAD, outflow graft obstruction

## Abstract

Left ventricular assist devices (LVADs) improve symptoms and outcomes in patients with advanced heart failure. We report the case of a patient with a freshly implanted HeartMate 3 LVAD, suffering abruptly on postoperative day 55 from pejoration of his heart failure with multiple episodes of low-flow alarm. Outflow graft obstruction (OGO) due to local aortic dissection was diagnosed with multimodality imaging. After a multidisciplinary discussion, a surgical approach was decided, and the patient benefited from a revision of his outflow graft.

Due to the worldwide increasing number of patients with advanced heart failure in the following decades the number of implanted left ventricular assist devices (LVADs) will also increase significantly.^1^ The differential diagnosis of low-flow alarms in LVAD patients is broad and includes tamponade, hypovolemia (bleeding), hypertension, arrhythmia, right ventricular failure, or outflow-/inflow/graft malfunction.^2,3^ Here, we report and discuss a case of early outflow graft obstruction (OGO) with acute heart failure decompensation after implantation of LVAD HeartMate 3.

## Case Report

### Initial Presentation

A 71 year old patient freshly implanted with a HeartMate 3 LVAD (Abbott, Chicago, IL) presented to the emergency department on postoperative day 55 with acute decompensated heart failure associated with multiple low-flow alarms from his device. The patient experienced a slow decline in functional capacity from New York Heart Association (NYHA) class II to III and a weight gain of 4 kilograms. Low-flow alarms progressively increased in number over time in the last few days, mainly during the morning hours.

Physical examination revealed normal LVAD mechanical sound with no clear auscultatory signs of pump thrombosis. New bilateral edema of the lower limbs and jugular vein distension were observed. The extremity was warm. Body temperature was 37.1°C, peripheral heart rate was 68 beats per minute, and blood pressure was 99/72 mm Hg. Oxygen saturation was 92% on room air. The origin of this patient’s severe heart failure (HFrEF) was a coronary artery disease (CAD) diagnosed following an acute STEMI infarct in 2014. Ten weeks before the presentation, the patient presented with an electrical storm with sustained monomorphic ventricular tachycardia (VT, cycle length 280 ms). The implanted defibrillator had delivered multiple adequate shocks. A coronary angiogram was performed, revealing a stable three-vessel CAD without acute occlusion or new high-grade stenosis. Ventriculography showed severely depressed systolic left ventricular function with an estimated ejection fraction below 20%. A provisory LVAD (pLVAD) with placement of an Impella (Abiomed, Aachen, Germany) via the right femoral artery was installed. An anti-arrhythmic therapy with amiodarone was started simultaneously. Initially, an attempt to ablate the VT under pLVAD support using a transseptal approach was decided and carried out on the third day of hospitalization. The scar region could be ablated, and ventricular support was successfully weaned the same day. During the subsequent hospitalization, the patient remained highly symptomatic (INTERMACS 3-4) despite the introduction of oral heart failure therapy. On the 11th day after admission, a durable HeartMate 3 LVAD was implanted as destination therapy (DT). The device implantation was done on standard sternotomy, and we used Prolen 4.0 suture for the graft anastomosis. We prepared the aorta with the on-pump beating heart technique. The aortic wall was stripped of fatty tissue and parts of aortic wall were excluded with an atraumatic Satinsky Debakey Clamp. After sharp incision followed a preparation of hole with 4.8 mm aortic punch (CleanCut, Quest Medical, Allen, Tx). After 27 days of hospitalization, the patient was discharged to the inpatient cardiac rehabilitation program.

### Investigations

**Figure 1.**
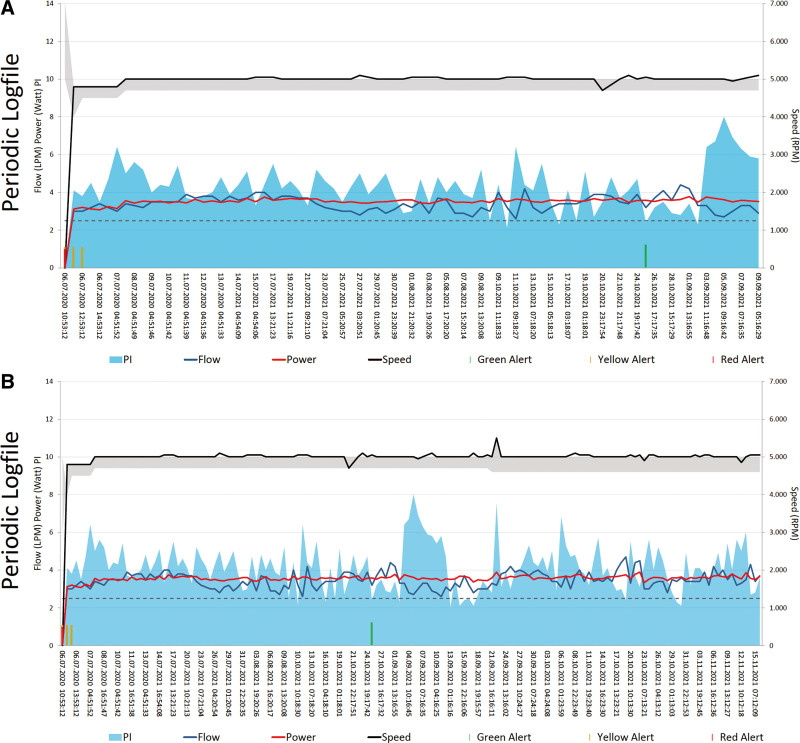
Logfiles. **A**: Multiple low-flow alarms with stable power and speed values. The pulse index was elevated by 8.0 during the events. **B**: Logiles after surgical revision with normalization of flow without alarms. LPM, liters per minute estimated flow; PI, pulsed index; RPM, revolutions per minute (speed).

**Figure 2.**
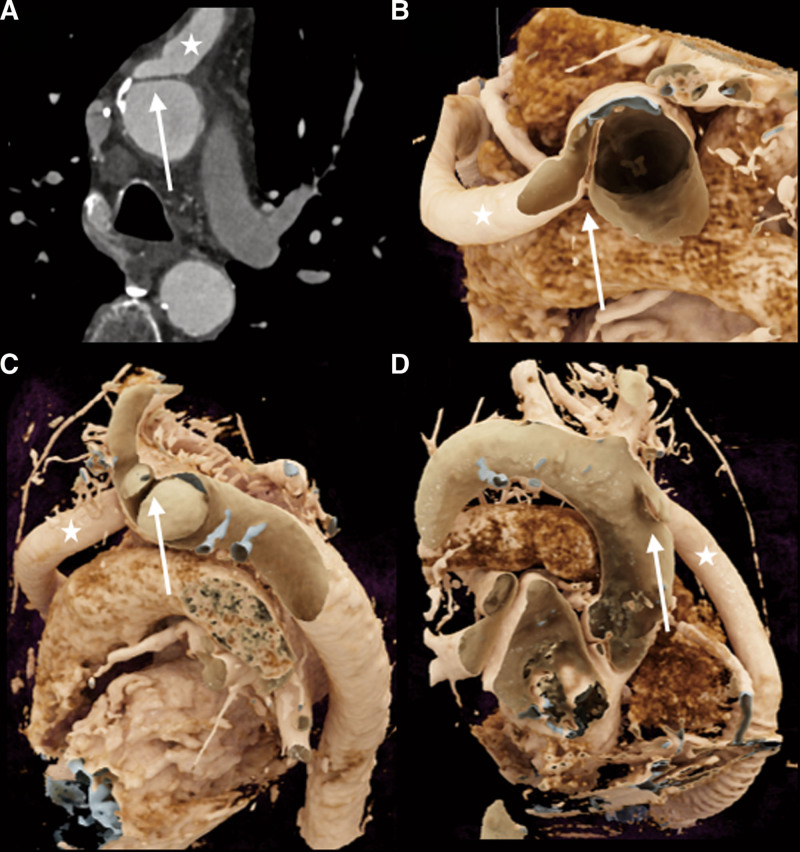
A cardiac computed tomography angiogram. **A–D**: chronic dissection (arrow) of the ascending aorta, directly in front of the orifice of the LVAD outflow graft (asterisk). LVAD, left ventricle assist device.

The 12-lead electrocardiogram (ECG) demonstrated sinus rhythm with a known complete left bundle branch block without signs of acute ischemia. Relevant laboratory results showed a significant increase in NTproBNP with a value over 19,000 pg/ml (baseline of 2,000–3,000 pg/ml) and acute kidney injury with an eGFR of 34 ml/min (baseline 45–50 ml/min). The hemoglobin level was stable at 130 g/L, and CRP was slightly elevated at 17 mg/L with a normal leukocyte count. INR International normalized Ratio (INR) was with 1.5, sub-therapeutic. Hemolysis parameters were within the normal range (negative). Log files showed multiple low-flow alarms during morning hours with stable power (3.6–3.8 watts) at a set speed of 5,000 rpm. The pulse index (PI) increased to 8 during the events, compared with the patient’s usual value (3–4) (Figure 1). Echocardiography revealed a small increase of LVEDD (58 *vs.* 55 mm) and no opening of aortic valve. Having ruled out the most common causes of low-flow alarms, the next step was to look for a pre-/post-LVAD obstruction. We proceeded with an ECG-gated computed tomography angiography (CTA) of the thorax and heart. Computed tomography angiography showed a thickened, chronic dissection of the ascending aorta originating at the anastomose between the LVAD outflow graft and the aorta ascendens (Figure 2A). The minimum diameter of the outflow graft anastomosis was 12 × 12 mm, with additional obstruction due to the dissecting membrane obstructing the opening (Figure 2, B–D). No other signs of occlusion or thrombosis of the inflow portion of the LVAD were found. An angiography to evaluate the possibility of a nonsurgical approach using a stent completed our investigations. The aortography confirmed the suspicion of local aortic dissection in the ascending aorta at the anastomosis site (Video 1A–B, Supplemental Digital Content). Surprisingly, up to this point, the invasive measurement showed no relevant gradient between the outflow graft and the ascending aorta. We therefore assumed that the obstruction after the initial dissection event was of dynamic nature.

**Video 1A.** 

**Video 1B.** 

### Initial Therapy and Short-Term Course

**Figure 3.**
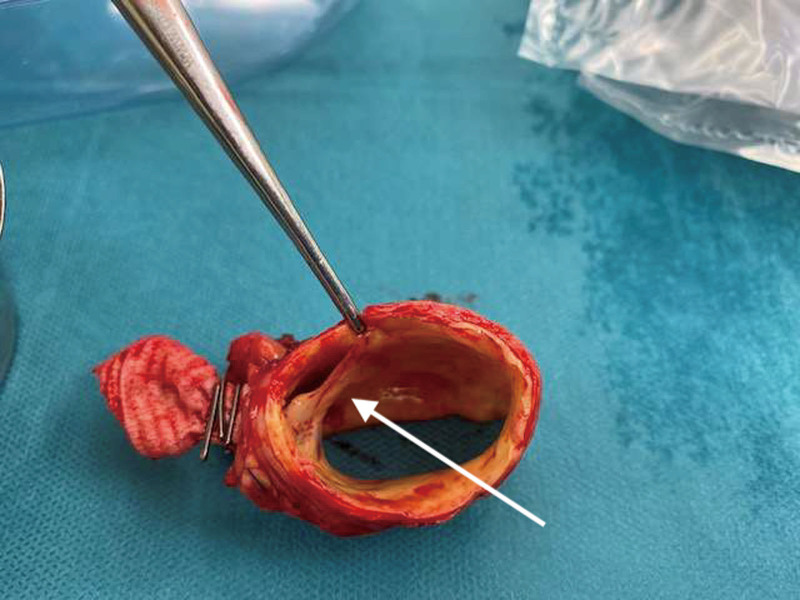
Intraoperative image. Segment of the dissected aorta with obstructing false lumen just under the intraoperatively clipped outflow graft anastomose (arrow), confirming CT images and angiogram. CT, computed tomography.

The local aortic dissection at the end-to-side anastomosis with a large reentry and obstruction of outflow graft could be confirmed during surgery and perioperative transesophageal echokardiography (TEE) (Figure 3 and Video 2, Supplemental Digital Content). Surgeons replaced the ascending aorta including the proximal arch and implanted a new outflow graft in deep hypothermic circulatory arrest and antegrade cerebral perfusion. The outflow graft was implanted in the usual fashion in the Dacron graft. In this case, the hole wasn’t prepared with the punch, but with a battery-operated cautery. The postoperative recovery was uneventful, with low-flow alarms disappearing completely.

**Video 2.** 

### Long-Term Course

Over the following months, the patient was seen regularly in our outpatient LVAD clinic. The patient’s NYHA and NTproBNP returned to baseline. The log files no longer show low-flow episodes; the PI has returned to its baseline value.

## Discussion

**Figure 4.**
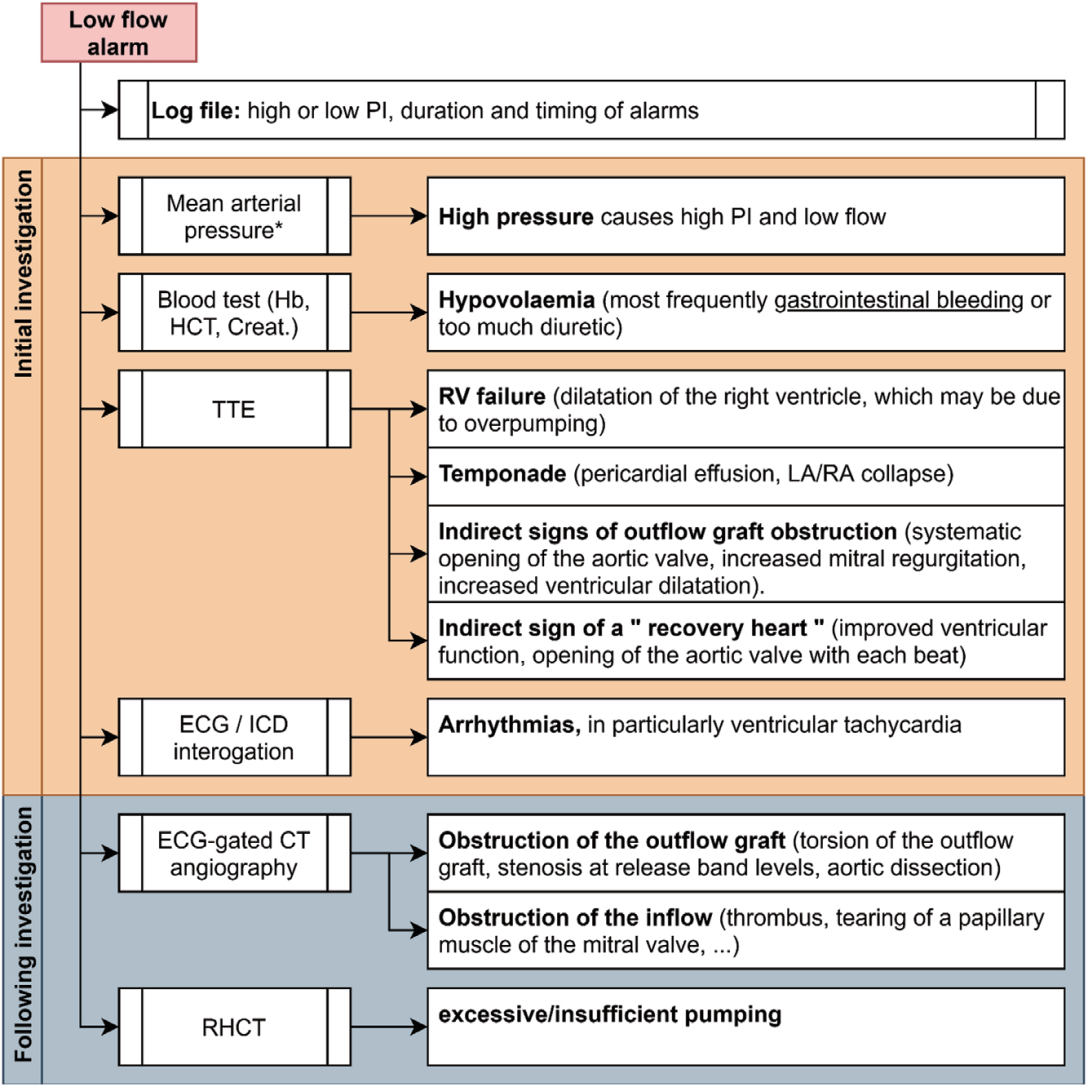
Proposed diagnostic algorithm of low-flow alarm. Creat, creatinine; ECG, electrocardiogram; Hb, hemoglobin; HCT, hematocrit; ICD, implantable cardioverter-defibrillator; LA, left atrium; PI, pulsed index; RA, right atrium; RHCT, right heart catheterization; RV, right ventricle; TTE, transthoracic echocardiography.

Implantation of LVAD either as a treatment until heart transplantation (BTT) or as DT increases survival and quality of life.^4^ During follow-up, parameters and alarms monitor the health status of the device. One of the most common alarms encountered during follow-up is the low-flow alarm. The diagnostic difference ranges from trivialities such as high blood pressure to life-threatening complications such as tamponade. There are various investigations available to diagnose the underlying cause, including LVAD interrogation (high or low PI), blood tests (hemolysis parameter and blood count), ECG (arrhythmia), and echocardiography. According to our diagnostic algorithm of low flor alarm, these investigations should be supplemented by CTA imaging if they appear negative (Figure 4).

Left ventricular assist device outflow graft obstruction is a rare complication (<1%) but is associated with significant morbidity and mortality. Outflow graft obstruction can be caused by external compression, twisting, or kinking of the outflow graft, accumulation of gelatinous protein between bend relief and outflow graft or thrombosis.^5^

Once a definitive diagnosis of OGO has been made, two treatment options are possible. Either a surgical approach with revision of the outflow graft or an endovascular approach with stenting.^6^ In our case, the stenting approach was technically not feasible given the stenosis’s location, so the surgical option was chosen.
